# Partner-directed gaze and co-speech hand gestures: effects of age, hearing loss and noise

**DOI:** 10.3389/fpsyg.2024.1324667

**Published:** 2024-05-30

**Authors:** Jeesun Kim, Valerie Hazan, Outi Tuomainen, Chris Davis

**Affiliations:** ^1^The MARCS Institute for Brain, Behaviour and Development, Western Sydney University, Sydney, NSW, Australia; ^2^Speech Hearing and Phonetic Sciences, University College London, London, United Kingdom; ^3^Department of Linguistics, University of Potsdam, Potsdam, Germany

**Keywords:** partner-directed gaze, co-speech gestures, hearing loss, aging, adaptation

## Abstract

Research on the adaptations talkers make to different communication conditions during interactive conversations has primarily focused on speech signals. We extended this type of investigation to two other important communicative signals, i.e., partner-directed gaze and iconic co-speech hand gestures with the aim of determining if the adaptations made by older adults differ from younger adults across communication conditions. We recruited 57 pairs of participants, comprising 57 primary talkers and 57 secondary ones. Primary talkers consisted of three groups: 19 older adults with mild Hearing Loss (older adult-HL); 17 older adults with Normal Hearing (older adult-NH); and 21 younger adults. The DiapixUK “spot the difference” conversation-based task was used to elicit conversions in participant pairs. One easy (No Barrier: NB) and three difficult communication conditions were tested. The three conditions consisted of two in which the primary talker could hear clearly, but the secondary talkers could not, due to multi-talker babble noise (BAB1) or a less familiar hearing loss simulation (HLS), and a condition in which both the primary and secondary talkers heard each other in babble noise (BAB2). For primary talkers, we measured mean number of partner-directed gazes; mean total gaze duration; and the mean number of co-speech hand gestures. We found a robust effects of communication condition that interacted with participant group. Effects of age were found for both gaze and gesture in BAB1, i.e., older adult-NH looked and gestured less than younger adults did when the secondary talker experienced babble noise. For hearing status, a difference in gaze between older adult-NH and older adult-HL was found for the BAB1 condition; for gesture this difference was significant in all three difficult communication conditions (older adult-HL gazed and gestured more). We propose the age effect may be due to a decline in older adult’s attention to cues signaling how well a conversation is progressing. To explain the hearing status effect, we suggest that older adult’s attentional decline is offset by hearing loss because these participants have learned to pay greater attention to visual cues for understanding speech.

## 1 Introduction

Typically, face-to-face spoken communication is interactive and adaptive, and uses information from multiple sources, i.e., from both the ears and eyes. Here, adaptation means that talkers alter their speech (both auditory and visual) based on of their perception of the needs of the interlocutor (Fitzpatrick et al., 2015; Hazan et al., 2018). Most research on how speakers adapt has simply examined speech signals, demonstrating effects of age (younger versus older adults, Hazan et al., 2018) and communicative conditions (Cvejic et al., 2012; Fitzpatrick et al., 2015; Smiljanic and Gilbert, 2017; Hazan et al., 2018). However, in a conversation there are other important communicative signals besides speech, signals that can convey speech related information (e.g., co-speech gestures, Drijvers and Özyürek, 2017), and those that can regulate aspects of a conversation (e.g., the frequency of partner-directed eye gaze, Degutyte and Astell, 2021). The aim of the current study was to develop a fuller picture of how talkers interact by examining these other communicative signals and determining whether these also show adaptation effects that differ as a function of age and communicative conditions. For the older adults, we also examined whether a participant factor that affects communication (mild hearing loss) played a role.

Before presenting the study in more detail, we first describe the functions of partner-directed gaze and co-speech gestures; why younger and older adults might differ in adjusting these behaviors; and what motivated the selection of the tested communication conditions. Partner-directed gaze likely serves a range of different functions based on perceiving information about the partner and signaling information to the partner. At a general level, it can provide and signal information about social/cognitive disposition. For example, Lindblom (1990) suggested that eye contact may be part of maintaining ‘communicative empathy’ with an interlocutor. In this regard, periodically maintaining visual contact with a conversational partner is likely an important part of feeling that one is *in* a conversation with that person. Indeed, it has been suggested that face-to-face conversation promotes expressiveness, social orientation and provides an attentional focus (Fish et al., 1993). More specifically, by looking at a partner, interlocutors can make eye contact. According to Rossano (2013), eye contact is important for scheduling turn-taking and pausing, as well as for understanding the attentional disposition of the interlocutor. As such, eye gaze is used to coordinate the timing of interlocutors’ contributions to the conversation (Kendon, 1967; Argyle and Cook, 1976; Bavelas et al., 2002), and to infer a partner’s current focus of attention (Hanna and Brennan, 2007). Moreover, it has been argued that eye contact is involved in modulating shared attention (Wohltjen and Wheatley, 2021) and mental state (Luft et al., 2022). In addition, partner-directed gaze furnishes information from the partner’s face and head motion, which contain information about speech (Kim et al., 2014) that enhances speech recognition (Sumby and Pollack, 1954; Davis and Kim, 2006).

**Figure 1:**
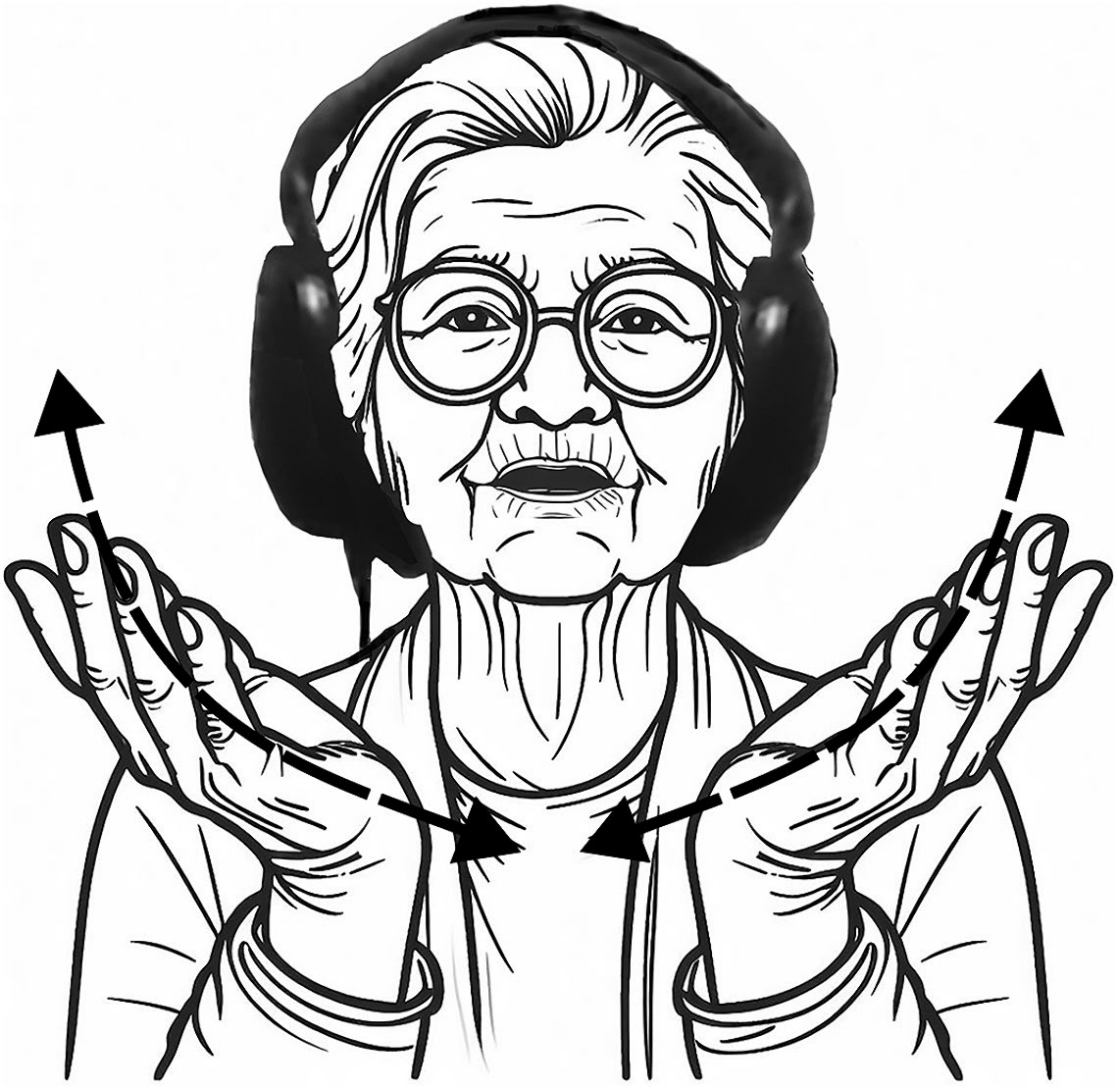
A depiction of an iconic co-speech gesture made by an older adult (HL) describing aspects of the Farm Scene version 2A. The talker said “there is a bowl on the table,” and at the same time made a curved gesture with her hands (palms-upward).

Co-speech hand gestures, gestures that occur with speech and convey information related to its content (Kita, 2023), also likely serve a range of functions both for speech perception and production. In the current study, we coded representational gestures, ones that had a semantic and temporal relationship with speech, rather than beat gestures (made for emphasis or to match/highlight some rhythm). The vast majority of these representational gestures were iconic, those that indicated the location, size shape of a specific referent, or depicted an action or type of movement of an object (see Figure 1 for an example). Given this, we have used the term iconic gesture throughout. It has been proposed that such co-speech gestures can assist speech comprehension (Kita et al., 2017), especially when speech is degraded. For example, it has been shown that seeing both visual speech and gesture boosts speech comprehension in noise to a level greater than visual speech alone (Drijvers and Özyürek, 2017). For speech production, two broad frameworks have been proposed, gesture for aiding conceptualization (Iverson and Goldin-Meadow, 1998; Krauss, 1998; Kita et al., 2017) and gesture as part of the action and perception system (Hostetter and Alibali, 2019). The latter framework has also been used to explain how seeing gestures may aid comprehension, i.e., seeing a gesture triggers a motor plan and subsequently a corresponding mental image that then aids comprehension (Hostetter and Alibali, 2019).

Given the linguistic and/or social functions of both partner-directed gaze and co-speech gestures, it is important to identify participant factors that may affect the extent of partner-directed gaze and co-speech gestures, respectively. One such factor is participant age, particularly with respect to displaying and gaining information about attentional disposition (of both the talker and listener). That is, there is evidence that older adults look at faces less often than younger adults. This behavior may be driven by age-related changes in picking up cues for social cognition (Grainger et al., 2019). Indeed, older adults appear less responsive to their conversational partner than younger adults. For instance, it has been shown that older talkers engage in ‘audience design’ less than younger talkers (Horton and Spieler, 2007). Likewise, older talkers tend not to adjust their narration (words and gestures) based on whether the content is old or new (Schubotz et al., 2015). In term of eye gaze specifically, older adults appear to be less sensitive to eye gaze as an attentional cue (McKay et al., 2022). Also, older adults tend not to look at the face to gain socially relevant information as much as younger adults (Grainger et al., 2019). For example, Vicaria et al. (2015) asked younger and older adults to rate the rapport of a person (target speaker) in videos of people having conversations. They found that older adults spent significantly less time looking at the target speaker’s head region compared to younger adults.

The evidence for a difference between older and younger adults in terms of using eye gaze to gain speech information is less clear. On the one hand, it has been shown that in face-to-face conversation, older adults fixate their social partner’s face less than young adults (De Lillo et al., 2021). However, this may not be the case for speech presented in noise or when the older adult has a hearing loss. As mentioned above, when speech is presented in noise, there is a substantial speech recognition benefit gained by looking at the speaker. Notably, it has been found that older adults get just as much benefit as younger adults, when noise is present (Tye-Murray et al., 2016). Moreover, the size of the perceptual benefit from seeing the talker was positively correlated with the degree of a participant age-related hearing problems (Puschmann et al., 2019). In sum, it may be that all talkers will increase the extent of partner-directed gaze in the presence of direct communication barriers such as noise or hearing loss, but older adults (with normal hearing) may not do this when only their partner experiences such a barrier. This prediction motivated the current study where we examined the frequency of partner directed gaze of younger and older talkers in different noise conditions, and also tested older adults with normal hearing and those with mild hearing loss.

The case of co-speech hand gestures has similarities with partner-directed gaze. Once again, there is evidence for a difference between younger and older adults, with older adults producing fewer iconic gestures than younger adults. For example, in a picture description task, Arslan and Göksun (2022) showed that younger adults produced a higher proportion of representational gestures than the older adults. In terms of perception, it appears that older adults may not benefit as much as younger adults from seeing co-speech gestures. That is, it has been found that older adults are worse than younger adults at integrating information from both speech and gesture (Cocks et al., 2011). Further, although viewing co-speech gestures provided some benefit for older adults’ speech perception in noise, this was less than what younger adults received (Schubotz et al., 2021). As far as we can tell, this work has not been extended to examine older adults with hearing loss. This is an important group to study with respect to co-speech gestures for several reasons. First, using gestures has been recognized as potential compensatory behavior for individuals with communication difficulties (Sparrow et al., 2020). Moreover, it has been proposed that people who routinely experience poor communicative situations in daily life automatically take gestures into account (Obermeier et al., 2012). Additionally, it has been suggested from epidemiological studies that older adults with hearing loss may be less adept in verbal fluency (Lin et al., 2011; Strutt et al., 2022). As mentioned above, one function of co-speech hand gestures is that they may aid conceptualization, thus it may be that older adults with hearing loss will use gestures to support lexical retrieval especially for demanding communication conditions, e.g., when there is a communication barrier. Given this, we would predict that any reduction in co-speech hand gestures that attends old age may be offset when talkers experience communication problems such as noise or hearing loss. To assess the impact of lexical retrieval problems, we include a measure of verbal fluency.

To gauge the extent of partner-directed gaze and co-speech hand gestures, the current study selected the DiapixUK task (Baker and Hazan, 2011). This task requires each member of a pair of participants to inspect a cartoon style picture and spot 12 difference by describing their pictures to the partner (one person has the role of primary talker, who takes the lead in the conversation). The Diapix task was chosen because it prompts on-going interactive communication. Further, it is a visually-oriented task that requires that talkers look at and note physical aspects of their pictures. This arrangement means that when partners look at each other they are taking ‘time-off’ from looking at and paying attention to their picture. Note that most of the studies that have examined partner-directed gaze have used a procedure or task that encourages mutual gaze. For example, in Luft et al. (2022) participants were explicitly instructed to look at each other’s eyes. Very few studies have examined partner-direct gaze in older and younger adults when a visually oriented task requires on-going interactive communication. The one study we know of that has used a joint verbal picture sorting task (similar to the DiapixUK) and found that younger adults gazed at their partners significantly more often than older adults did (Lysander and Horton, 2012). This result suggests that a visual task is appropriate for revealing age differences in partner directed communication behavior (e.g., gaze and gesture).

In designing the current study, we took into account that talkers can adjust their communicative behaviors to help themselves (self-oriented) and/or to meet their partner’s need (partner-oriented). While the former case is expected to occur when talkers experience communication difficulties (Sparrow et al., 2020), the latter is expected to occur when talkers are aware that listeners have communication difficulties (see the Hyper-hypo theory of Lindblom, 1990). It is not clear whether the self-oriented adjustments would differ between age groups, but the partner-oriented ones may occur less in older adults. This is because at a broad level, it has been suggested that older adults might suffer declines in basic social perception (Slessor et al., 2008) and in joint attention (Erel and Levy, 2016). As such, older adults may be less aware of their partner’s communicative needs. Given this, the current study was designed to distinguish between adaptations that occur as a response to the noise itself and partner-oriented responses by examining what occurs when both the primary (talker A) and the secondary talker (Talker B) are in noise versus when only Talker B is in noise (i.e., the talker A does not experience the communication barrier).

In sum, the current study examined the frequency and duration of partner-directed gaze and frequency of co-speech hand gestures in spontaneous speech communication task as a function of age, hearing loss and different types of barriers to communication. The effect of age was examined by contrasting the performance of younger adults with that of older adults. The effect of hearing loss was tested by selecting two groups of older adults, those who had age-related hearing loss (older adult-HL) and those who did not, i.e., who had relative normal hearing (older adult-NH). Note, although including young adults with hearing loss would create a fully balanced 2×2 factorial design, this group was not included as age-related hearing loss, rather than hearing loss alone, was the variable of interest given the focus on aging and communicative compensation strategies. The effect of adaptation was tested by using four communication conditions. The first was a no barrier (NB) condition in which the primary and secondary talkers heard each other unobstructed. The effect of partner-oriented adaptation was tested in two conditions that reduced the ability of the secondary talker to hear the primary talker’s speech. In one condition, the secondary talker had a background of babble speech noise (BAB1), this would impede hearing but also induce auditory and visual Lombard speech, i.e., a phenomenon whereby a talker alters their vocal and visual speech production in noisy environments, (Lane and Tranel, 1971; Kim et al., 2005) when the secondary talker spoke. In the other condition, the secondary talker experienced a simulated hearing loss (HLS). In the final condition both talkers spoken and listened in a background of babble speech (BAB2).

Based on the studies briefly reviewed above, we would expect that in general, younger adults would gaze at their partners and produce co-speech hand gestures more frequently than older adults, although this behavior may be modulated by hearing loss. Studies of partner-directed gaze and gesture suggest that these behaviors increase for conversations held in noise (BAB2); although the increase in gesture was in the kinematics, not frequency of occurrence (Trujillo et al., 2021). It is unclear what to expect for the two partner-oriented conditions (BAB1 and HLS). However, if a change in behavior in these conditions (compared to the NB one) requires that Talker A picks up social cues that their partner is struggling (Slessor et al., 2008; Erel and Levy, 2016) and adjusts their behaviors accordingly (e.g., to meet their partner’s need), then it may be that older adults will show less of a change than younger adults since they may not pick up the partner cues. Once again, this may be modulated by hearing loss.

## 2 Method

### 2.1 Participants

Fifty-seven single-sex pairs of native Southern British English adult talkers between the ages of 19 and 84 years participated in the study. Participants had no self-reported history of speech or language impairments. All older adults passed the shorter version of the mini-mental state examination (MMSE) dementia screening (>18 out of maximum 20). The pairs consisted of 57 “primary” talkers (Talker A participants) and an additional 57 “secondary” talkers (Talker B participants), who acted as conversational partners with Talker A participants but whose speech was not analyzed. The secondary talkers were always young adults (aged between 18 and 30 years); were the same sex as Talker A, and all passed a hearing screen at 25 dB HL or better at octave frequencies between 250 and 8,000 Hz in both ears.

**Figure 2:**
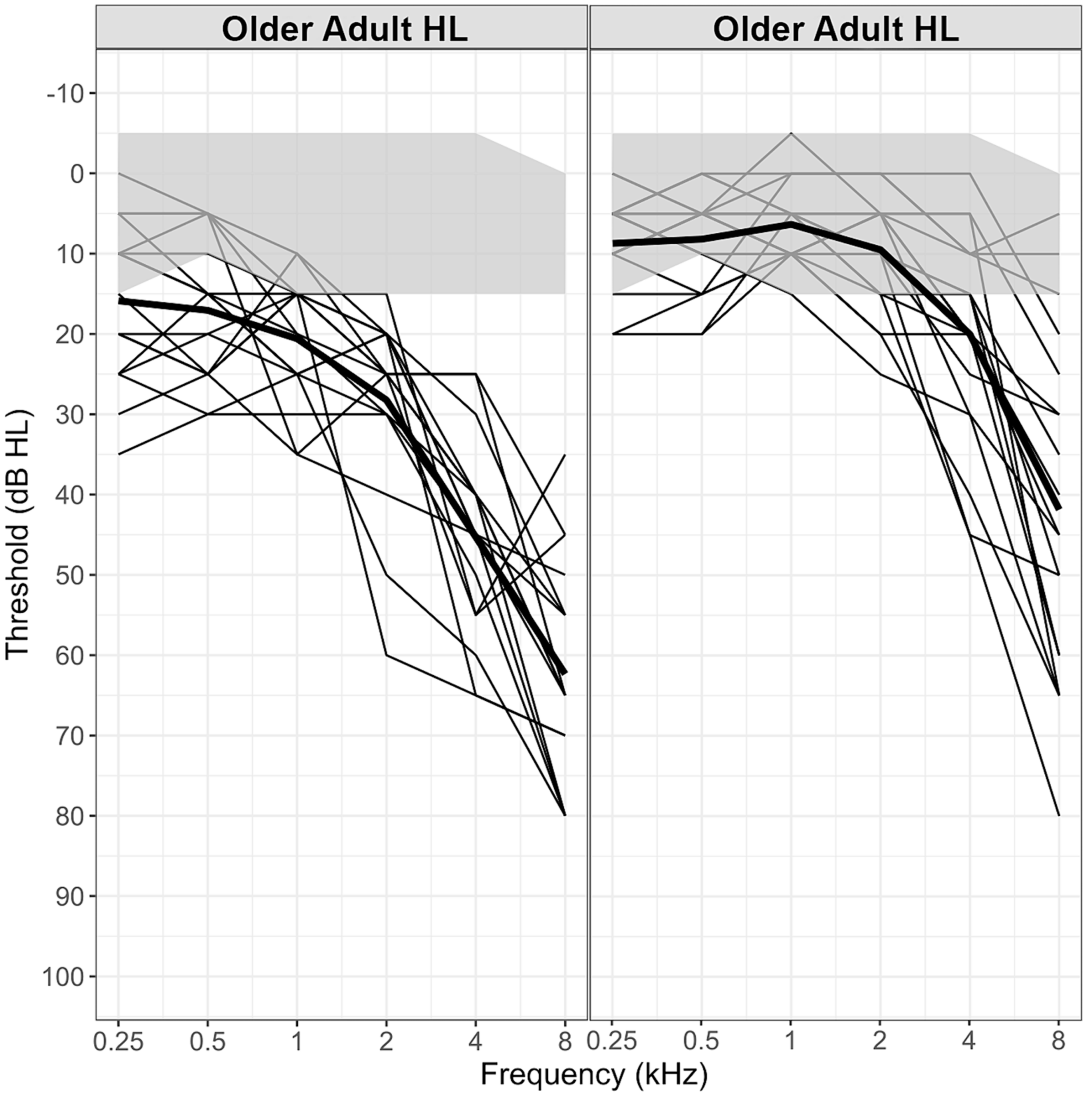
Better ear Hearing Level scores for the older adults with mild hearing loss (older adult-HL) and the older adults with relatively normal hearing (older adult-NH). The gray region shows the boundaries of the Younger Adult scores. Thin black lines show each participant’s scores, the solid black lines indicate the mean.

The 57 primary talkers (Talker A participants) consisted of three participant groups divided in terms of age and hearing capacity. An older adult group with mild (aging related) Hearing Loss group (older adult-HL): *N* = 19 (11 female; M female-age = 72.4 years, M male-age = 75.8 years). Participants had an average hearing threshold of <45 dB between octave frequencies 250–4,000 Hz, with a symmetrical downward slope of pure tone threshold in the high frequency range typical for an age-related hearing loss profile. An older adult normal hearing group (older adult-NH): *N* = 17 (12 female; M female-age = 70.1 years, M male-age = 73.6 years). Participants had a hearing threshold of <25 dB between octave frequencies 250–4,000 Hz. A younger adult normal hearing group (YA): *N* = 21 (13 female; M female-age = 21.5 years, M male-age = 20.5 years). Participants had a hearing level of 25 dB or better at octave frequencies between 250 and 8,000 Hz in both ears. A summary of the hearing level data id shown in Figure 2. As can be seen, the hearing levels of the older adult-HL group diverge markedly from those of both the normally hearing older and younger adults. To establish whether the difference in hearing levels for the older adults affected speech recognition, thresholds of word intelligibility in background noise were measured. This was done using the WiNics task (Hazan et al., 2009) that was modeled on the coordinate response measure (Moore, 1981). The older adults with mild hearing loss (older adult-HL) required a significantly less masking (SNR −5.6 dB) for threshold performance (79.4%) than the older adults with normal hearing (SNR −6.5 dB), *t*(31.5) = 2.07, *p* = 0.471.

### 2.2 Materials and tests

The experimental task was the DiapixUK, a “spot the difference” picture task. The DiapixUK consists of 12 picture pairs that belong to one of three themes, beach, farm, and street scenes.^1^ Additional tests were run to assess cognitive and linguistic abilities. Both short-term and working memory were evaluated using digit span tests. Short-term memory was tested using a forward digit span (DSF) task; working memory with a backward digit span (DSB) test that measures information storage and rehearsal. In these memory span tasks, the participant repeated auditorily presented number sequences in the same or reverse order and were scored as correct or incorrect for each sequence (maximum scores, DSF = 16 and DSB = 14). There were two trials for each span (starting with 2 digits). The test stopped when both trials were failed. The number of correct trials passed were counted. In addition, the efficiency of lexical search and retrieval was measured using a verbal fluency task in which participants had to say as many words as possible from a category in 60 s; the final score for this test was the total number of items across the three categories.

### 2.3 Procedure

**Figure 3:**
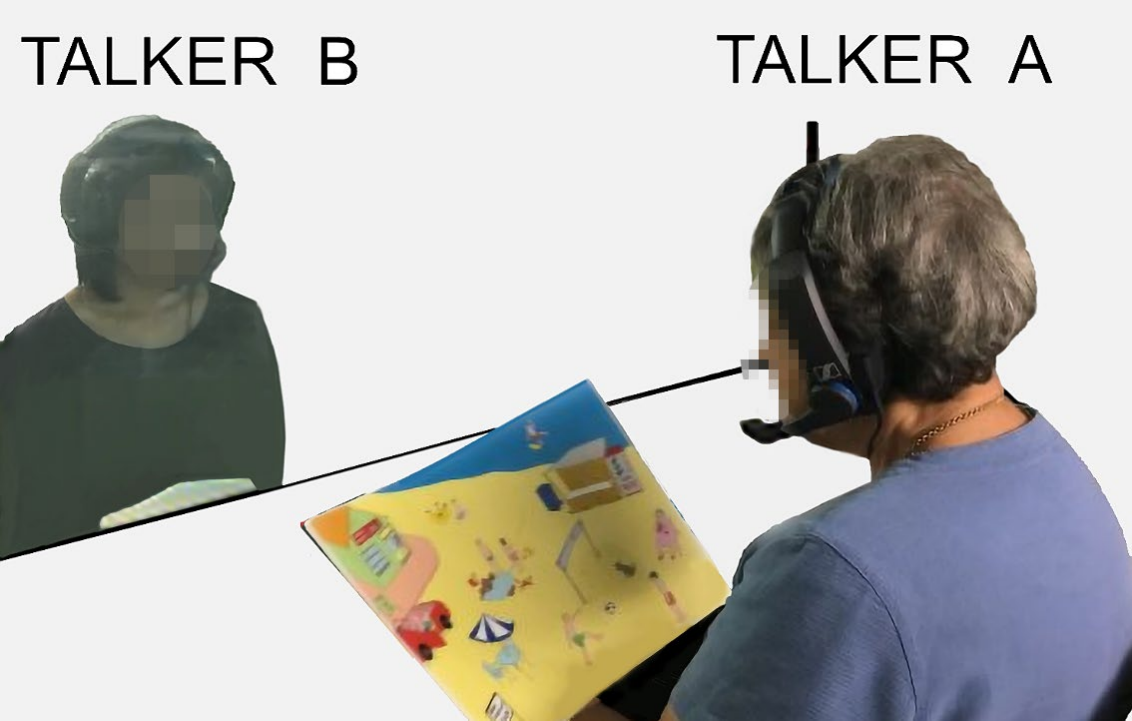
A schematic depiction of the interaction setup. Participants wear headsets and hear each other under different listening conditions.

Participants were tested in pairs, with each seated in different room (see Figure 3). Each participant was assigned the role of a primary talker (‘Talker A’) or a secondary talker (‘Talker B’). The primary talker was instructed to take the lead and to do most of the talking. Older adults were always primary talkers (Talker A). Younger adults were either primary or secondary talkers.

As mentioned, only younger participants were assigned the role of Talker B (the productions of these talkers were not analyzed). This was done in order to hold one aspect of the partner relatively constant. Choosing only younger adults was based on the study by Vandeputte et al. (1999). Vandeputte et al. showed that both younger and older participants exhibited a higher level of social skill, as measured by the composite partner attention score, when paired with younger adults than when paired with older adults.

Note that participants did not know the interlocutors that they were paired with. Studies have found that participants who are familiar with each other gaze at each other more than unfamiliar pairs (e.g., Beattie, 1980). Given that it would be difficult to equate familiarity, it was decided to pair people who did not know each other.

**Figure 4:**
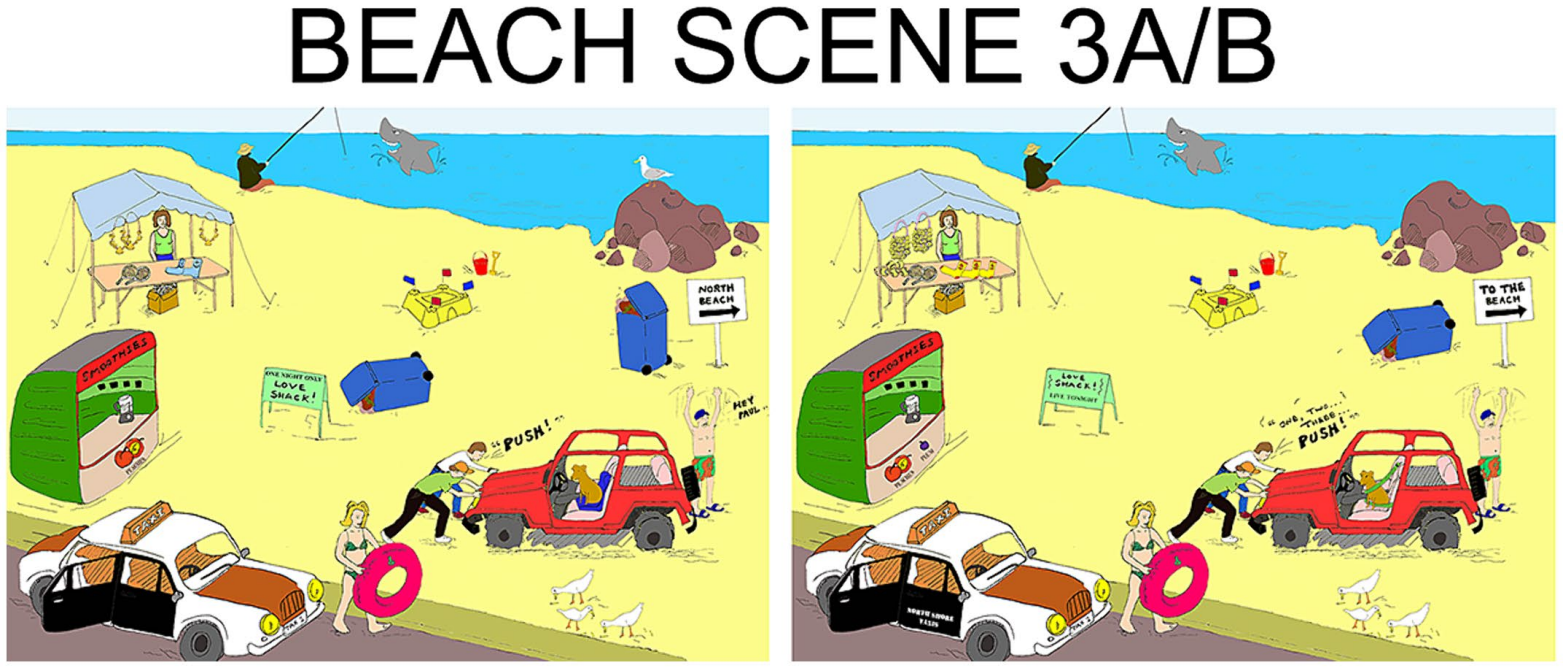
One of the 12 pairs of pictures from the DiapixUK set, each pair contained 12 differences [(Hazan and Baker, 2011), CC BY 4.0].

In the task, participants were given a different picture from the DiapixUK set (see Figure 4) for each communication condition. Participants were instructed that the pictures contained 12 differences and that their task was to collaborate in conversation to discover these. They had a time-limit of 10 min per picture.

Before commencing the experimental proper, all participants had a practice session that lasted until they had identified 6 differences from a different set of pictures. For this practice session, participants were seated in the same room.

Participants completed the task under four communication conditions consisting of a ‘No Barrier’ (NB) and three barrier conditions (BAB1, HLS, and BAB2). In the NB condition, both talkers heard normally, i.e., in quiet. In the BAB1 condition, Talker B heard Talker A in 8-talker (4 female, 4 male), babble noise (from Cooke and Lu, 2010). The SNR for the BAB1 condition was individually set using an adaptive procedure to equate performance for the HLS condition (see below) on the Modified Rhyme Test (MRT). In the HLS, ‘Hearing Loss Simulation’ condition, Talker B heard Talker A via a real-time hearing loss simulator modeling a profound sensorineural loss at levels 40–50–60–90 dB at frequencies 250–500–1,000–4000–8000 Hz; (HeLPS, the Hearing Loss and Prosthesis Simulator, Zurek and Desloge, 2007). That is, speech to Talker B was delivered in a manner that simulated severe-to-profound hearing loss. In the BAB2 condition, both talkers heard each other in the 8-talker babble noise at 0 dB SNR. In the HLS and BAB2 conditions automated gain control was employed to achieve a signal to noise ratio (SNR) of 0 dB. This gain control meant that speaking louder would not affect the SNR for Talker B and was employed so that there would be a similar communication barrier in the conditions. Since the NB was the easiest condition, participants were always given it first, with the order of the three barrier listening conditions randomized. In the experiment, Talker A was informed about what their listening partner was hearing, i.e., that in BAB1 that they were listening in noise, or in the HLS condition that they would be experiencing a simulated hearing loss. Talker A did not experience these conditions directly. Auditory and video recording were made of Talker A. An Eagle G157b lapel microphone was used for the auditory recording with a 640 × 480 (VGA) camera at 30 fps for the video (that captured Talker A’s head and upper body, see Figure 4).

### 2.4 Data processing

The video recording (of Talker A) for each condition was annotated using the ANVIL editor (Kipp, 2014). Annotation consisted of marking the time of occurrence of events that occurred in the video. An annotator marked when the talker raised their head to look at their conversational partner and also when an iconic hand gesture was made. An example iconic co-speech gesture is shown in Figure 1. The onset and offset times of the marked event were recorded, and an annotation comment appears overlayed on the video stream to enable a quick review of marker placement (confirmed by another observer). The data from ANVIL was used to compile an event map for a particular behavior. The event data was used to calculate the total number of events (in this case, number of times Talker A looked at their partner and used an iconic co-speech hand gesture) and the sum of the duration of gaze events (i.e., the mean total partner-directed gaze time).

## 3 Results

A preliminary analysis of the two older adult groups was conducted using the data of the two memory span tasks. For the forward digit span results i.e., the number of trials (see above), there was no significant difference in the scores of the older adult-HL (*M* = 11.82) and the older adult-NH (12.00) groups, *t*(1,29.5) = 0.259, *p* = 0.797, Bayes factor for the null model = 3.2. This also the case for the backward digit span results, older adult-HL (*M* = 7.06) and older adult-NH (*M* = 7.59), *t*(1,30.8) = 0.734, *p* = 0.469, Bayes factor for the null model = 3.203. There was also no significant difference between the older adult groups and the younger adult group, for forward digit span, the Bayes Factor for the null model = 6.871; for backward digit span = 5.142. These analyses suggest that any subsequent differences between groups are unlikely to be due to differences in short-term or working memory.

Before examining the gaze and gesture data, we first examined if the time taken to complete the Diapix task (Overall duration) differed between the participant groups (older adult-HL, older adult-NH, younger adult) and the communication conditions (NB, BAB1, HLS, and BAB2), and whether there was an interaction between these two variables. A mixed design ANOVA (using the r afex package, Singmann et al., 2021) was conducted (with Participant group a between subjects factor and Communication condition, a within subjects factor); Model: aov_car (Overall duration ~ Participant group*Communication condition + Error) (Participant group/Communication condition). The difference in task duration between the participant groups was not significant (older adult-NH, *M* = 466 s, SE = 15.8; older adult-HL, *M* = 482 s, SE = 12.1; younger adult, *M* = 434 s, SE = 15.8), *F*(2, 45) = 0.99, *p* = 0.378. There was a significant overall effect of communication condition, *F*(2.81, 126.38) = 8.82, *p* < 0.001, with the NB (*M* = 454.5 s, SE = 18.01), BAB1 (*M* = 444.68 s, SE = 16.40) and BAB2 (*M* = 428.74 s, SE = 15.91) having similar completion times, and the HLS condition taking more time (*M* = 513.84, SE = 15.83). The interaction between these variables was not significant, *F*(5.62, 126.38) = 1.99, *p* = 0.076. In brief, there was no significant difference in the time it took participants to complete the Diapix task (find 12 differences) between the three participant groups (older adult-HL, older adult-NH, younger adult) and no significant interaction effect on completion time between communication condition and participant group.

Two aspects of the partner-directed gaze data were analyzed, the number of times Talker A looked at their partner (gaze frequency) and the sum of the duration of gaze events (gaze duration). The frequency of iconic co-speech hand gestures was also analyzed. In what follows we first report the gaze frequency data and then the gaze duration data. Following this we present the hand gesture data.

### 3.1 Partner-directed gaze

#### 3.1.1 Gaze frequency

Since the gaze frequency data consisted of counts of events, we used a poisson mixed model (estimated using ML and Nelder–Mead optimizer) to predict gaze number as a function of Communication condition (NB, BAB1, HLS, BAB2) and Group (older adult-HL, older adult-NH, younger adult), formula: (gaze number ~ Communication condition x Group). The model included Participant as random effect (formula: ~1 | Participant). The model was run using the afex r package (Singmann et al., 2021). Note that attempting to generate maximal or near maximal models (e.g., add in random slopes to the random variable) led to failures to converge, thus we accepted a simpler model, rather than risk the problems associated with fitting overparameterized models (see Matuschek et al., 2017).

**Figure 5:**
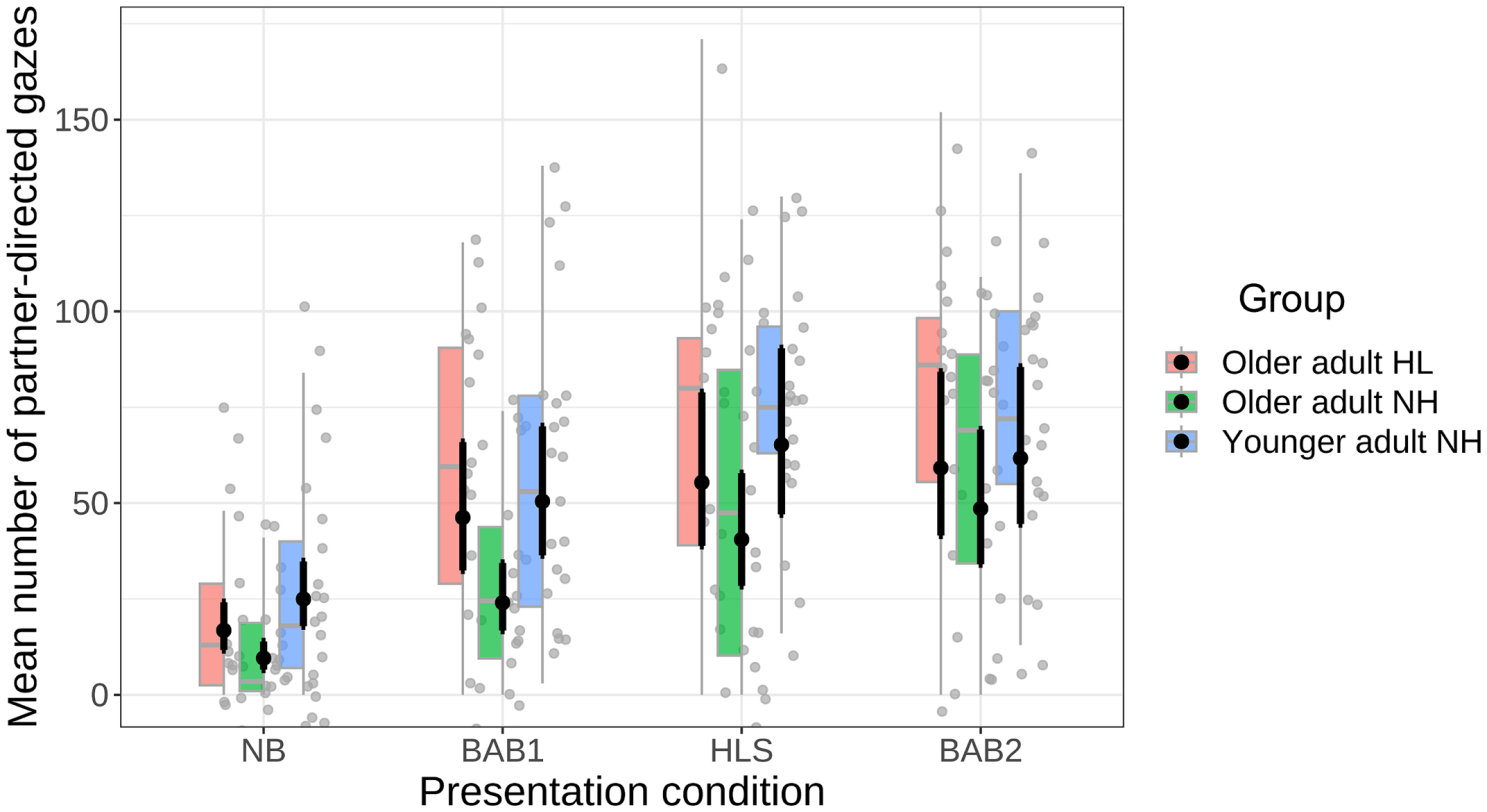
Mean number of partner-directed gaze events as a function of Communication condition and Participant Group. Boxplots are color coded, the gray dots are participant data (jittered); black circles show the mean and black whiskers show model-based standard error.

Figure 5 shows the mean total gaze number (partner-directed gaze) for the three participant groups as a function of communication condition (plotted using afex_plot, Singmann et al., 2021). As can be seen, the mean number of partner-directed gazes differed across the communication conditions; NB condition (*M* = 21.26, SE = 3.23); BAB1 (*M* = 49.75, SE = 4.96); HLS (*M* = 66.11, SE = 5.13); BAB2 (*M* = 69.56, SE = 4.90). The effect of Communication condition was significant, ${Χ}^{2}$ = 1904.50, *p* < 0.0001; as was the effect of Group (older adult-NH, *M* = 38.81, SE = 4.27; older adult-HL, *M* = 55.90, SE = 4.98; younger adult, *M* = 58.95, SE = 4.12), ${Χ}^{2}$ = 6.03, *p* = 0.049 and the interaction between these variables, Communication condition x Group, ${Χ}^{2}$ = 117.69, *p* < 0.0001.

Planned statistical significance tests were conducted using the emmeans package (Lenth, 2021) with *p*-values adjusted using the Holm method; and the results are shown in Table 1. As can be seen in the table, for the NB condition there was a significant difference in the number of partner-directed gazes as a function of hearing status, with older adult-NH (*M* = 12.1, SE = 3.81) exhibiting fewer than older adult-HL (*M* = 23.8, SE = 5.62). There was also a significant effect of age, with older adult-NH making fewer partner directed gazes than younger adult (*M* = 28.64, SE = 6.25).

**Table 1:** Summary of the outcome of the planned comparisons for number of partner-directed gazes as a function of Hearing status and Age (younger adult, YA; older adults, OA; normal hearing, NH; hearing loss, HL).

Communication condition	Testing	Contrast	Z-ratio	*p* value
NB	Hearing status	OA-NH vs. OA-HL	−2.114	0.035*
	Age	OA-NH vs. YA	−3.752	0.0002**
BAB1	Hearing status	OA-NH vs. OA-HL	−2.541	0.011*
	Age	OA-NH vs. YA	−2.990	0.0028**
HLS	Hearing status	OA-NH vs. OA-HL	−0.934	0.350
	Age	OA-NH vs. YA	−1.633	0.102
BAB2	Hearing status	OA-NH vs. OA-HL	−0.777	0.437
	Age	OA-NH vs. YA	−0.977	0.329

*Indicates statistical significance at *p* < 0.05; **at *p* < 0.01.

A similar result was found for the BAB1 condition. That is, a significant difference for hearing status, with older adult-NH (*M* = 30.39, SE = 5.86) making fewer gazes than older adult-HL (*M* = 58.56, SE = 9.10); and a significant effect of age with older adult-NH making fewer gazes than younger adult (*M* = 58.8, SE = 8.84). It is also clear, that the number of partner-directed gazes increased in the BAB1 compared to the NB condition (a post-hoc test confirmed a significant difference between the conditions, ${Χ}^{2}$ = 620.53, *p* < 0.0001).

Although the patterns of mean values for the participant groups in the HLS and BAB1 conditions were similar, the statistical analysis outcomes in the former condition were not. That is, the effects of hearing status and age were not significant in the HLS condition. Two things are apparent from Figure 5 concerning the HLS condition. The first is that, overall, there was a greater number of partner-directed gazes in the HLS compared to the BAB1 condition (a post-hoc test confirmed a significant difference between the conditions, ${Χ}^{2}$ = 162.09, *p* < 0.0001). The second is that there appears to be greater variability in the number of gazes for the older adult-NH group compared to the other groups. We will return to these points of difference in the Discussion. The final contrast was for the BAB2 condition, where both talkers spoke and listened in 8 talker babble noise. As can be seen in Table 1, neither the effects of Hearing status nor Age were significant.

#### 3.1.2 Gaze duration

**Figure 6:**
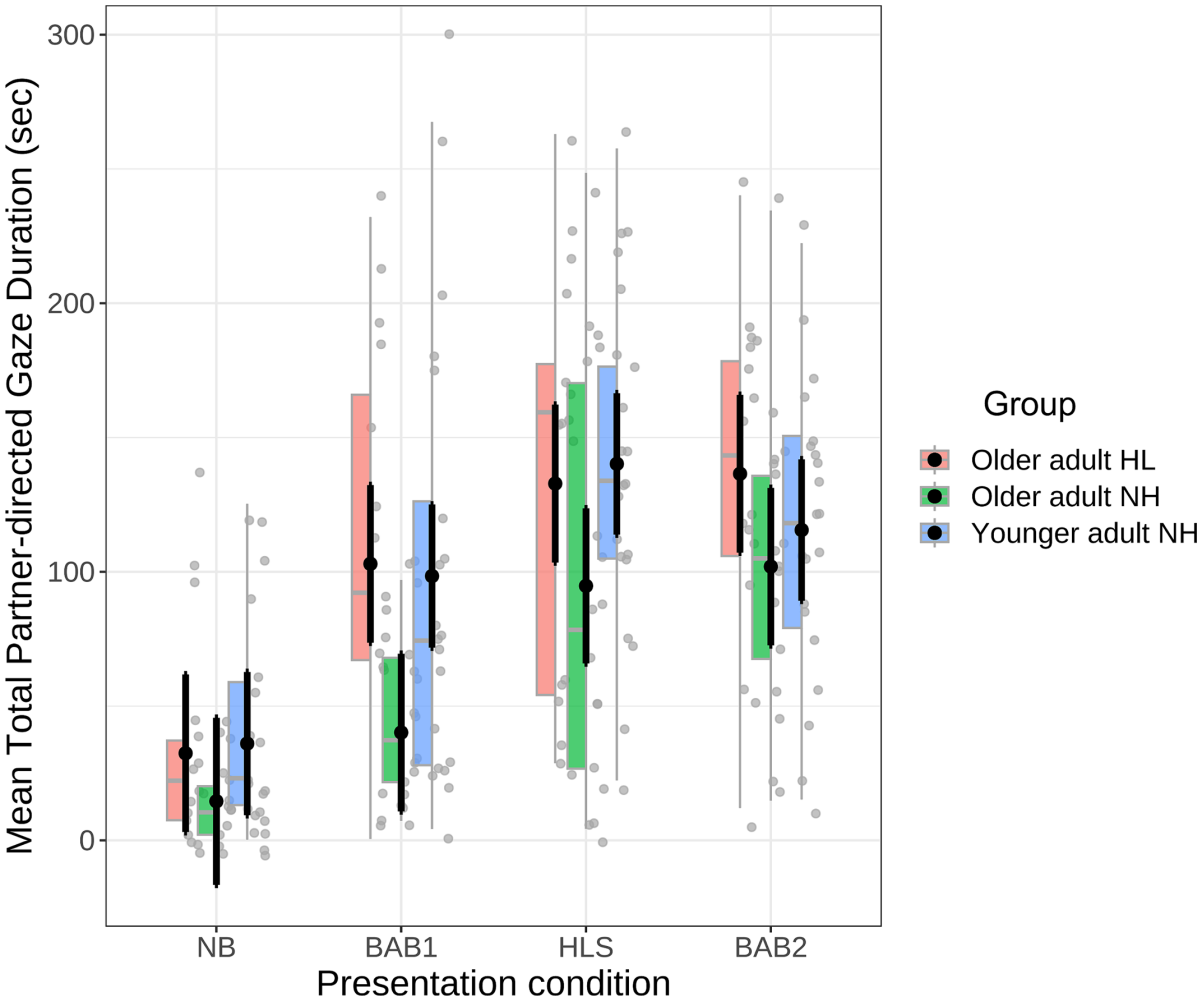
Mean total partner-directed gaze time as a function of Communication Condition and Participant Group.

In addition to the number of times that Talker A gazed at Talker B, we also tested for differences in mean total looking time as a function of Communication condition (NB, BAB1, HLS, BAB2) and Group (older adult-HL, older adult-NH, younger adult). That is, a person could gaze less often at their conversational partner, but each gaze could be of a longer duration. To examine this, the total looking time for each participant in each communication condition was determined; these data are summarized in Figure 6.

As can be seen in the figure, there was a difference in the gazing duration across the communication conditions, (NB, *M* = 27.7 s, SE = 8.48; BAB1, *M* = 80.5 s, SE = 8.29; HLS, *M* = 122.6 s, SE = 8.22; and BAB2, *M* = 118.0 s, SE = 8.26). The mean total gaze duration data was analyzed by fitting a linear mixed model using the afex r package (Singmann et al., 2021) to predict Gaze Duration with Communication condition and group as fixed factors (formula: Duration ~ Condition * Group) and Participant as a random effect (formula: ~1 | Participant). Note, adding random slopes to Participants led to the model failing to converge.

There was a significant overall effect of communication condition, *F*(3,143.092) = 62.66, *p* < 0.001. The overall effect of participant Group was not significant, *F*(2,52.861) = 3.07, *p* = 0.054, nor was the interaction between communication condition and group, *F*(6,143) = 1.73, *p* = 0.117. For consistency with the gaze frequency analysis, the same planned contrasts between conditions were conducted using the emmeans r package (Lenth, 2021). The results of these analyses are shown in Table 2.

**Table 2:** Summary of the outcome of the planned comparisons for gaze duration as a function of Hearing status and Age (younger adult, YA; older adults, OA; normal hearing, NH; hearing loss, HL).

Communication condition	Testing	Contrast	Z-ratio	*p* value
NB	Hearing status	OA-NH vs. OA-HL	−0.828	0.408
	Age	OA-NH vs. YA	−1.040	0.299
BAB1	Hearing status	OA-NH vs. OA-HL	−3.000	0.004**
	Age	OA-NH vs. YA	−2.914	0.004**
HLS	Hearing status	OA-NH vs. OA-HL	−1.833	0.067
	Age	OA-NH vs. YA	−2.305	0.021*
BAB2	Hearing status	OA-NH vs. OA-HL	−1.651	0.099
	Age	OA-NH vs. YA	−0.686	0.493

*Indicates statistical significance at *p* < 0.05; **at *p* < 0.01.

Table 2 shows that for the NB condition the difference in the mean duration of partner directed gazes as a function of hearing status was not significant, although the direction of the difference in means was in the expected direction with mean gaze duration older adult-NH (*M* = 15.84, SE = 4.71) less than older adult-HL (*M* = 36.55, SE = 10.88). The effect of Age was also not significant.

The results for gaze duration in the BAB1 condition was very similar to that found for gaze frequency. That is, there was a significant effect of Hearing status (older adult-NH vs. older adult-HL) and also a significant effect of Age (older adult-NH vs. younger adult). In both cases, the older adults with normal hearing looked at their conversation partner for less time than the comparison group. The results for the HLS condition were similar to those for the gaze frequency data, with the exception that for gaze duration, there was a significant effect of Age. That is, older adults with normal hearing looked at their partners for less time than the younger adults. Finally, there were no significant effects of hearing status or age in the BAB2 condition.

### 3.2 Co-speech hand gestures

**Figure 7:**
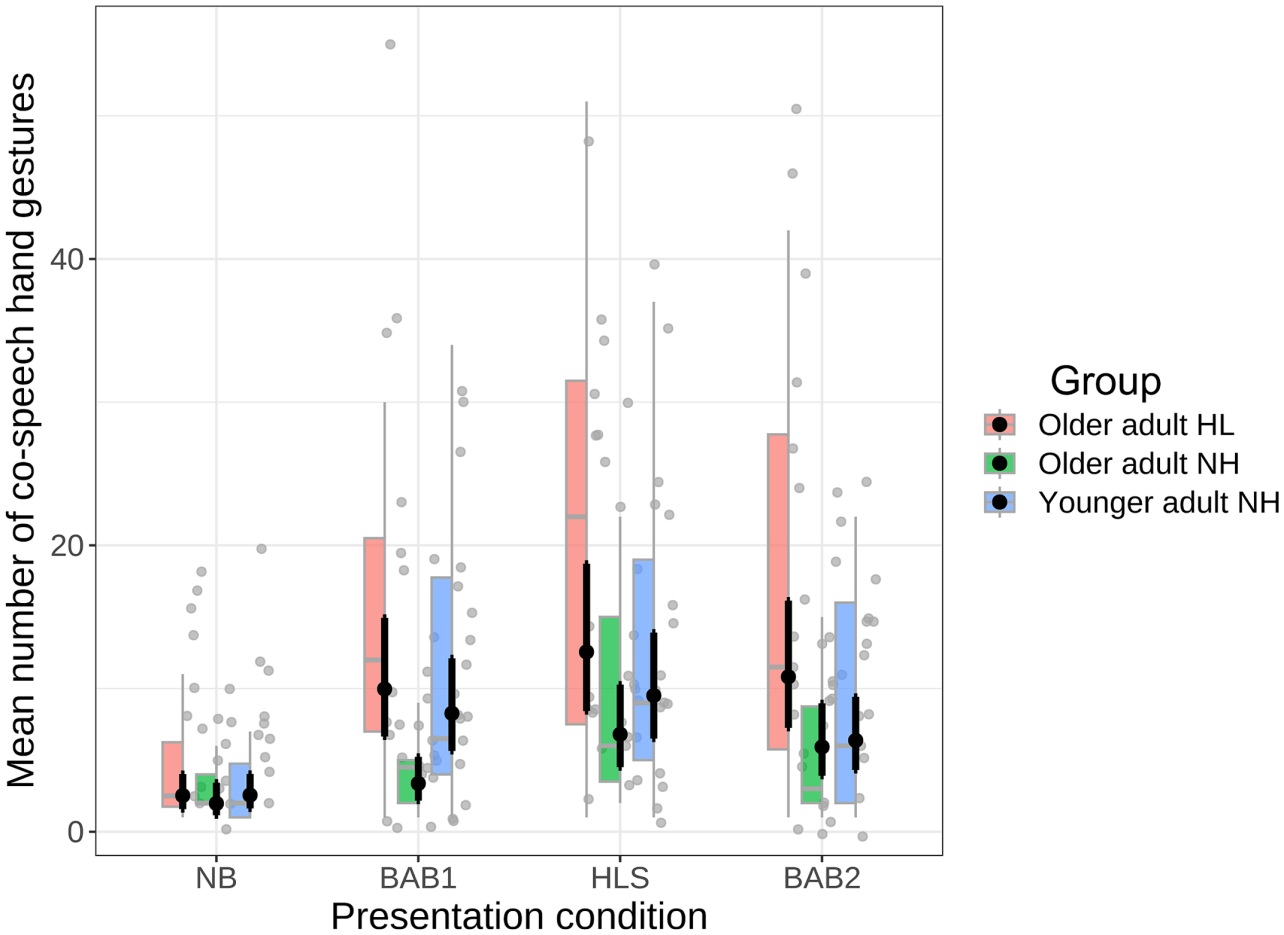
Mean number of co-speech hand gestures as a function of Communication condition and Participant Group.

The same analyses as were conducted for partner-directed gaze were carried out for Talker A’s co-speech hand gestures. The mean number of iconic co-speech hand gestures as a function of Communication condition and participant group is shown in Figure 7. As can be seen in the figure, the overall pattern of co-speech hand gestures across participant groups and conditions was very similar to that of the partner-directed gaze data. As with the gaze data, we used a poisson mixed model to the number of gestures as a function of Communication condition (NB, BAB1, HLS, BAB2) and Group (older adult-HL, older adult-NH, younger adult), formula: (gesture number ~ Communication condition × Group). The model also included Participant as random effect (formula: ~1 | Participant).

The analysis showed that mean number of iconic co-speech gestures differed across the communication conditions: NB condition (Mean = 3.79, SE = 0.61); BAB1 (Mean = 10.83, SE = 1.69); HLS (Mean = 14.40, SE = 1.80); and BAB2 (Mean = 0.96, SE = 1.61). The effect of Communication condition was significant, ${Χ}^{2}$ = 232.91, *p* < 0.0001. The effect of Group was not significant (older adult-NH, Mean = 6.20, SE = 0.82; older adult-HL, Mean = 15.23, SE = 1.93; younger adult, Mean = 10.06, SE = 1.20), ${Χ}^{2}$ = 4.76, *p* = 0.09. There was a significant interaction between these variables, Communication condition x Group, ${Χ}^{2}$ = 26.66, *p* < 0.001.

The same paired comparisons as were conducted for the gaze data above were carried out on the gesture data, again using the emmeans package (Lenth, 2021) with *p*-values adjusted using the Holm method. The results are shown in Table 3.

**Table 3:** Summary of the outcome of the planned comparisons for co-speech hand gestures as a function of Hearing status and Age (younger adult, YA; older adults, OA; normal hearing, NH; hearing loss, HL).

Communication condition	Testing	Contrast	Z-ratio	*p* value
NB	Hearing status	OA-NH vs. OA-HL	−0.664	0.5068
	Age	OA-NH vs. YA	0.709	0.4785
BAB1	Hearing status	OA-NH vs. OA-HL	−3.567	0.0004**
	Age	OA-NH vs. YA	−3.026	0.0025**
HLS	Hearing status	OA-NH vs. OA-HL	−2.098	0.0359*
	Age	OA-NH vs. YA	−1.173	0.2409
BAB2	Hearing status	OA-NH vs. OA-HL	−2.053	0.0401*
	Age	OA-NH vs. YA	−0.255	0.7988

*Indicates statistical significance at *p* < 0.05; **at *p* < 0.01.

From Table 3 it can be seen that for the NB condition there was no significant effect of Hearing status or Age. The results for the BAB1 condition were very similar to the gaze results. That is, there was a significant effect of Hearing status (older adult-NH vs. older adult-HL) and also a significant effect of Age (older adult-NH vs. younger adult). In both cases, the older adults with normal hearing made fewer partner co-speech gestures than the comparison group. The results for the HLS condition showed that there was a significant effect of Hearing status, with older adults with normal hearing making fewer gestures than older adults with hearing loss.

### 3.3 Verbal fluency and the gaze and gesture behaviors

Having outlined specific patterns of partner-directed eye gaze and co-speech hand gestures across the participant groups and communication conditions, we briefly consider how these behaviors were associated with the conversation task more generally, and whether the number of gestures was influenced by verbal fluency (as suggested in Krauss, 1998; Obermeier et al., 2012; Sparrow et al., 2020).

For the general analysis relating to conversation task, we looked at the Hearing status contrast (older adult-NH and older adult-HL) and the Age contrast (older adult-NH and younger adult) by probing the relationship between the total time that the partners spoke (to complete the task) as a function of number of eye gazes, number of co-speech hand gestures, and participant group for each on the communication barrier conditions (BAB1, HLS, BAB2). For this we employed three linear regression model, formula lm(duration ~ Gaze number + Hand gesture number + Hearing status), one for each barrier condition. The results for the hearing status contrast analysis are shown in Table 4. The analysis results for the Age group contrast are shown in Table 5.

**Table 4:** Hearing status contrast: summary of the linear regression analyses predicting overall task duration as a function of number of partner-directed gazes, number of co-speech hand gestures and Hearing status (older adult-NH vs. older adult-HL) for each communication condition.

Condition	Variable	*F* value	*p* value
BAB1	Gaze number	4.855	0.037*
	Hand gesture number	0.670	0.421
	Hearing status	0.956	0.338
HLS	Gaze number	5.142	0.031*
	Hand gesture number	0.366	0.55
	Hearing status	1.227	0.277
BAB2	Gaze number	0.082	0.777
	Hand gesture number	4.306	0.047*
	Hearing status	0.652	0.426

*Indicates statistical significance at *p* < 0.05.

**Table 5:** Age contrast: summary of the linear regression analyses predicting overall task duration as a function of number of partner-directed gazes, number of co-speech hand gestures and Age (older adult-NH and younger adult) for each communication condition.

Condition	Variable	F value	*p* value
BAB1	Gaze number	15.308	0.0005**
	Hand gesture number	0.549	0.4649
	Age	1.514	0.2284
HLS	Gaze number	3.597	0.069
	Hand gesture number	2.272	0.144
	Age	4.095	0.0534
BAB2	Gaze number	0.215	0.647
	Hand gesture number	4.828	0.03645*
	Age	1.483	0.233

*Indicates statistical significance at *p* < 0.05; **at *p* < 0.01.

The results in Table 4 can be summarized as following. When Talker A can clearly hear Talker B, who themselves face a barrier in hearing Talker A (BAB1 or HLS), the number of Talker A’s partner-directed gazes is a significant predictor of the duration of the task. However, when Talker A also experiences a barrier, then it is the number of co-speech gestures that Talker A makes that is a significant predictor of the task duration. We did not test for interactions between the factors, as such comparisons are underpowered (see Brysbaert, 2019); however, for the older adult-HL group in the BAB1 and HLS conditions, the correlation between number of gazes and overall task duration was not significant (*R* = 0.076, *p* = 0.71; *R* = 0.03, *p* = 0.91, respectively). Interestingly, there was a significant correlation the older adult-HL group between number of hand gestures and overall task duration in the BAB2 condition (*R* = 0.56, *p* = 0.018).

As can be seen in Table 5, the pattern of results for the two different age groups (older adult-NH and younger adult) was similar to that of the Hearing status groups (Table 4). That is, in the BAB1 condition the number of partner gazes was significantly associated overall time; and for the BAB2 condition, the number of gestures had a significant association with overall time. For the HLS condition, none of the effects achieved the traditional statistical significance level of 0.05.

The data from the verbal fluency task were analyzed using in independent t-test between the older adult-HL and older adult-NH groups. The results showed that the older adult-HL group produced significantly fewer category instances than the older adult-NH one, *t*(33.9) = −3.54, *p* = 0.0012. To examine the relationship between the verbal fluency scores and number of hand gestures for older groups (combined data), we used Pearson correlations for each of the barrier conditions (BAB1, HLS, BAB2). There were significant negative correlations (i.e., the fewer category instances produced the more hand gestures) for each condition, BAB1, *R* = −0.38, *p* = 0.042; HLS, *R* = −0.38, *p* = 0.037, BAB2, *R* = −0.4, *p* = 0.022. For comparison, for the younger adult data, none of the comparisons between the verbal fluency scores and hand gestures were significant, BAB1, *R* = 0.152, *p* = 0.56; HLS, *R* = 0.28, *p* = 0.33; BAB2, *R* = −0.12, *p* = 0.68.

## 4 Discussion

The study examined visually oriented adaptations (eye gaze and co-speech hand gestures) made by adult talkers when communicating with a conversational partner in a quiet and three challenging communication barrier conditions. In general, compared to the no barrier condition, in the barrier conditions both the number and duration of partner-directed gaze and iconic co-speech gestures increased. However, this increase was not uniform across the participant groups and the communication barrier type. That is, talkers adapted these non-speech communicative signals to communication conditions, while the degree of such adaptation was modulated by age, hearing status and communication condition.

Before discussing the specific condition, age, and hearing status comparisons, we first consider the task, that is, how the task related to partner-gaze and gesture, and to one aspect of participant cognitive ability (i.e., short-term memory). First, it is worth emphasizing that the Diapix is a conversation-based joint problem-solving task that does not require that participants look at each other or to use hand gestures. Whereas looking at an interlocutor (or gesturing) is important for a range of functions in a basic face-to-face conversation (e.g., coordinating joint behavior; signaling information about attention, and so on), it was less clear how often such behaviors would occur in the visually-oriented Diapix task (e.g., Argyle and Graham, 1976). We used this task based on the rationale that if partner gaze and co-speech gestures did occur it would indicate that these behaviors were in some way important for communication. Here, the term ‘important’ need not apply solely to task performance, but could reflect broader aspects of communication that go beyond solving specific task based problems (see Broader implications, below).

In terms of the relationship between task time and gaze and gesture behavior, the most straightforward assumption is that the longer the task goes on, the more such behaviors should occur. Task duration cannot be the sole factor modulating these behaviors since duration did not differ across the participant groups, and there was no significant communication condition by group interaction. Nevertheless, for the hearing contrast groups, and the age contrast groups overall, there was a positive relationship between Diapix task time and number of partner-directed gazes, and task time and iconic co-speech gestures, i.e., significant effects for the number of eye gazes for the BAB1 condition, and number of co-speech hand gestures for the BAB2 condition. What should be noted, however, is that for the older adult-NH group alone, this was not the case. That is, there was no significant correlation between number of eye gazes and task time in the BAB1 (*R* = 0.07) or HLS conditions (*R* = 0.03). By contrast, the correlations for the younger adult group for these conditions were substantial (*R* = 0.69 and 0.46). In all, we suggest that communication difficulties resulted in an increase in both task time and gaze and gesture behavior. Hence, task time and the amount of gaze and gesture behavior tended to be correlated particularly in difficult communications. Of course, because this relationship is underpinned by gaze behavior (instrumental for picking up partner signals), if Talker A is less able to attend to these signals, then there would be no such correlation. Indeed, older adult-NH did not increase their gaze or gesture behaviors in the BAB1 compared to the NB condition (see below).

One additional point about the task is worth considering. In their task-based conversation study, Lysander and Horton (2012) found that older adults had fewer partner-directed gazes than younger adults. To explain this result, they proposed that older adult looked at their partner less due to a decline in short-term memory interacting with task demands. That is, they proposed that because older adults had to pay more attention to the visual matching component of their task, they had less time to look at their partner. Their results were consistent with this idea as the number of partner-directed gazes was correlated with the older adult’s short-term memory span. Although this may have been the case for their particular task, there was no such correlation in the current study, and no difference between the older adult-HL and older adult-NH groups in short-term or working memory as indicated by the digit span forward/backward results, or between the younger adult scores and the older adult ones.

### 4.1 Age and partner-directed gaze

We measured the effects of age on the number and duration of partner-directed gaze by contrasting the older adult-NH and younger adult groups. In what follows, we briefly discuss the age effects we found for each of the communication conditions.

In the no barrier condition, there was an effect of Age for the number of partner-directed gazes, with the older adult-NH group producing fewer than the younger adult group. The older adult-NH result is consistent with the idea that older adults are less sensitive to communicative cues from the interlocutor because they tend not to look at the face to gain socially relevant information as much as younger adults (Vicaria et al., 2015; Grainger et al., 2019; De Lillo et al., 2021).

The difference between the older adult-NH group and the younger adult group was clearest for the BAB1 condition, where Talker B heard Talker A’s speech in babble noise. Here, the number of partner-directed gazes by the older adult-NH group was similar to the NB condition, and once again may have been due to a disposition to not attend to communicative cues. The reason why the age effect increased was because the number of partner-directed gazes in the younger adult group was much greater than in the NB condition. This increase was likely in response to cues from Talker B indicating that understanding Talker A’s speech was effortful. That is, because Talker B listened in babble noise their responses were likely more forced both auditorily and visually (e.g., Lombard speech, Lane and Tranel, 1971; Kim et al., 2005) than in the NB condition, and this attracted the younger adult Talker A’s attention. This idea is consistent with an observational study by Skelt (2010) that suggested a talker’s speech disfluencies and gestures can act as gaze soliciting signals.

There was no Age effect for the HLS condition for the number of partner-directed gazes but there was for gaze duration, with older adult-NH looking for a significantly shorter time at their conversational partner than the younger adult group. The lack of a significant difference for the count data appears to be due to the greater variability in the number of eye gazes produced by the older adult-NH group. The degradation in the HLS condition would have led to clearer signaling by Talker B that they found it difficult to understand what was being said. That is, the hearing loss simulator modeled a profound sensorineural loss. This type of hearing degradation would have been novel to younger adults (Talker B) whereas the background multitalker babble speech (BAB1) would not have been. As such, they may have experienced more difficulty in adapting to it and hence greater comprehension difficulty in HLS than BAB1. Some of older adult-NH participants would have picked this up and subsequently increased the number of their gazes to monitor this.

In the BAB2 condition, there was no significant difference in the number of partner-directed gazes between the older adult-NH and younger adult groups. A reason for this is that in this condition Talker A also listened in noise, so there was likely more self-oriented behavior to assist with speech perception. That is, Talker A simply looked at their partner to gain visual speech information (Mixdorff et al., 2007).

### 4.2 Hearing status and partner-directed gaze

The effects of Hearing status was measured by contrasting the data from older adults with normal hearing to that from older adults with hearing loss. The basic findings were similar to the Age contrast, with the older adult-NH group showing fewer eye gazes than the comparison group (in this case the older adult-HL group) for the NB and BAB1 conditions (count data) and only the BAB1 condition for the duration data. In explaining the effect of age, we proposed that older adults may have an age-related decline in attending to cues from their partner; such a decline would also need to be assumed to have occurred for the older adult-HL group. This leaves open the question of why the older adult-HL group looked more often at their partner in the NB condition, and increased their partner-directed gazes in the BAB1 condition. This increase must have been in response to what Talker B did in the BAB1 condition (as Talker A did not experience a barrier just like the NB condition). One possibility is that older adult-HL are more used to problems arising in speech communication and so routinely look at their interlocutor for cues (and hence looked more often than the older adult-NH even in the NB condition). In the BAB1 condition, Talker B may have signaled their difficulty hearing by relatively subtle face cues, something picked up by the older adult-HL group (but not by the older adult-NH one) and prompting them to monitor their partner more.

### 4.3 Age and co-speech gestures

In a recent review chapter on older adult gesture, Göksun et al. (2022) concluded that in comparison to younger adults, older adults used fewer co-speech gestures in spontaneous discourse. We found this pattern in the BAB1 condition, but not in the NB condition, likely because too few iconic co-speech gestures in the younger adult comparison group to be able to pick up any difference. To explain the effect of Age, we once again assume that the reduction in number of co-speech gestures for older adult-NH vs. the younger adult group was due to age-related changes in picking up cues for social cognition. In addition, the younger adult group showed an increase in gestures in the BAB1 condition. Here, we assume that the younger adults picked up cues from their interlocutor indicating that they had difficulty understanding, and this motivated an increase in gestures.

As mentioned above, in the HLS condition Talker B experienced a hearing loss simulation that modeled a profound sensorineural loss. This is a barrier to hearing that young adults most likely would not have experienced; leading them to produce more signals indicating their hearing difficulty, so that even some in the older adult-NH picked this up and increased their gestures to assist.

Once again for the BAB2 condition, in which Talker A was also speaking in noise, there was no group difference. This is consistent with the general idea that when an external factor, such as noise, has a large influence, it will be less likely that participant factors play a role. Evidence for the influence of noise in the BAB2 condition comes from contrasting the number of gestures the older adult-HL group made when there was no noise (BAB1) to when there was (BAB2, see Figure 6). As can be seen, the older adult-NH participants made more gestures when they were in noise (BAB2) compared to when only their partner was in noise (BAB1). This result is consistent with past research that gesture frequency increases under degraded listening conditions (Kendon, 2004) and also shows that the older adult-NH do use co-speech hand gestures under some conditions.

### 4.4 Hearing status and co-speech gestures

The effect of Hearing status in the no barrier condition was not significant. This null finding suggests that whether older adult-HL participants gesture more than their older adult-NH counterpart depends upon whether their conversational partner signals that they are experiencing hearing problems. That is, if Talker B has no hearing barrier, they would not experience any difficulty in hearing Talker A and so would not produce any cues that they have any problems. Thus, even though the older adult-HL participants look more often at the partner in the NB condition than the older adult-NH group (see above), this additional visual monitoring would not lead to additional gesturing.

In the BAB1 condition there was a significant effect of Hearing status (older adult-NH vs. older adult-HL). That is, older adults with normal hearing (older adult-NH) made fewer partner co-speech gestures than the older adult-HL group. To explain this difference, we again suggest that older adult-NH exhibit fewer communicative gestures due to age-related changes in picking up cues for social cognition. For the older adult-HL group, we make the additional suggestion that due to their hearing problems they looked more at their interlocutor (Talker B), who because they were in noise produced cues indicating their hearing difficulty, and these in turn spurred the older adult-HL group to use more gestures.

However, if the older adult-HL gestures are driven by cues picked up from Talker B, then why does Talker A’s verbal fluency score correlate with the number of their gestures? One way that this might occur is if Talker A noticed that Talker B was struggling and wanted to help. If Talker A was able to quickly retrieve words, they would not need to gesture, but if Talker A’s lexical retrieval was slow, then they may have gestured to assist their own lexical retrieval. A related idea that could explain the relationship with verbal fluency comes from the Gesture as Simulated Action framework (Hostetter and Alibali, 2019). Here, gesture production is conditioned by such factors as the activation of the producer’s motor system, and the readiness of the producer to perform a gesture (gesture threshold). If people with poorer verbal fluency have a lower gesture threshold, then they may be more ready to gesture when they observe that Talker B is having difficulties. Gesture patterns in the HLS and BAB2 conditions for the older adult-HL group follow those of the eye gaze data; something to be expected if gesture is, at least, in part responsive to information picked up by viewing the conversational partner.

Throughout we have presumed that older adult-HL may be used to looking at their communication partners because of their hearing loss. That is, they give greater weight to visual cues for understanding speech or have become more sensitive to the visual speech related cues because this is useful in their daily lives. However, this may not be the case if they often used hearing aids. As it turned out, this was not the case since information from a background questionnaire indicated that although some of the older participants owned hearing aids, all but one reported either not using them at all or only using them very occasionally.

### 4.5 Broader implications

We have found that in some conditions, older adults without hearing loss looked less frequently and for less time at their conversational partners and gestured less compared to younger adults. We proposed that this result may be due to a decline in older adult’s attention to cues signaling how well a conversation is progressing. If so, what might be a consequence of being less sensitive to, and not producing observable behaviors that match a partner’s communicative needs? Although beyond what was measured in the current study, research has found that one of the reasons that young adults express dissatisfaction with the conversations of older adults is due the perception that they have to over accommodate to the older partner to make up for their perceived underaccommodation (Harwood and Williams, 1998). Furthermore, Gasiorek and Dragojevic (2017) examined what happens when underaccommodation occurs over time. They showed that not only is the communication of a person who repeatedly underaccommodates rated as poor but that the person themselves is less favorably evaluated.

It may seem that since the older adult-HL group are behaving like younger adults, at least with respect to mutual eye gaze and gestures, they should receive the benefits that such interaction bestows (i.e., give a good impression of attentiveness). If this were the case, does it represent an ‘upside’ to hearing loss? We do not think so. It should be pointed out that this group of older adult-HL are active and socially involved, as well as motivated to achieve. This predisposition may have propelled this group to make an extra effort to communicate based on cues picked up by their putative disposition to attend to their partner’s facial and hand gestures. Evidence that supports the extra effort hypothesis comes from the analysis of the auditory data, where we found the older adult-HL group displayed indicators of vocal effort (Hazan et al., 2019). That is, unlike the other groups (younger adult and older adult-NH), there was a correlation for the older adult-HL group between increases in median F0 and mid-frequency speech energy, a hallmark of increased vocal effort (Hazan et al., 2018). This strategy of increased effort adopted by the older adult-HL group is likely to increase both vocal strain and mental fatigue.

## 5 Conclusion

The current results showed older adults with normal hearing produced fewer partner-directed gazes than younger adults when there was no barrier to communication. Moreover, when their interlocutors had listening problems (BAB1) the number of partner-directed gazes and gestures by the older adults remained at the level of the no barrier condition while for younger adults both increased. These age effects are likely due to changes in how older adults deploy attention. This is because when the older adults themselves experienced a communication barrier (BAB2) they showed similar amounts of gaze and gesture as the younger adults. Also, older with hearing loss did not show this decrement in gaze/gesture behavior. That is, older adults can increase gaze and gesture to their partner in response to challenging conditions, but this may occur only when they are predisposed to do so. Indeed, it is worth noting that more older adults with normal hearing looked and gestured with a partner who experienced an unfamiliar communication barrier (the HLS condition) rather than a familiar one (BAB1). We suggest that this was due to the partners experiencing greater problems in the unfamiliar HLS condition thus producing more overt signals of communication breakdown, which some of the older adults with normal hearing picked up. In all, our findings highlight the possibility that older adults with normal hearing may show decreased sensitivity to an interlocutor’s communicative problems but that this is not necessarily something that is fixed.

## Data availability statement

The datasets presented in this study can be found in online repositories. The names of the repository/repositories and accession number(s) can be found at: https://osf.io/4rwxq/.

## Ethics statement

The studies involving humans were approved by the UCL Research Ethics Committee. The studies were conducted in accordance with the local legislation and institutional requirements. The participants provided their written informed consent to participate in this study.

## Author contributions

JK: Conceptualization, Methodology, Writing – original draft. VH: Conceptualization, Methodology, Project administration, Supervision, Writing – review & editing. OT: Conceptualization, Methodology, Writing – review & editing. CD: Conceptualization, Formal analysis, Methodology, Writing – original draft.

## Footnotes

^1^see https://zenodo.org/record/3703202 where all materials are available.
